# A comparative study of the nanoscale and macroscale tribological attributes of alumina and stainless steel surfaces immersed in aqueous suspensions of positively or negatively charged nanodiamonds

**DOI:** 10.3762/bjnano.8.205

**Published:** 2017-09-29

**Authors:** Colin K Curtis, Antonin Marek, Alex I Smirnov, Jacqueline Krim

**Affiliations:** 1 Department of Physics, North Carolina State University, Raleigh, NC 27695, USA; 2 Department of Chemistry, North Carolina State University, Raleigh, NC 27695, USA

**Keywords:** additives, alumina, aqueous colloids, fractal, friction, lubricants, nanodiamond, nanotribology, quartz crystal microbalance, stainless steel

## Abstract

This article reports a comparative study of the nanoscale and macroscale tribological attributes of alumina and stainless steel surfaces immersed in aqueous suspensions of positively (hydroxylated) or negatively (carboxylated) charged nanodiamonds (ND). Immersion in −ND suspensions resulted in a decrease in the macroscopic friction coefficients to values in the range 0.05–0.1 for both stainless steel and alumina, while +ND suspensions yielded an increase in friction for stainless steel contacts but little to no increase for alumina contacts. Quartz crystal microbalance (QCM), atomic force microscopy (AFM) and scanning electron microscopy (SEM) measurements were employed to assess nanoparticle uptake, surface polishing, and resistance to solid–liquid interfacial shear motion. The QCM studies revealed abrupt changes to the surfaces of both alumina and stainless steel upon injection of –ND into the surrounding water environment that are consistent with strong attachment of NDs and/or chemical changes to the surfaces. AFM images of the surfaces indicated slight increases in the surface roughness upon an exposure to both +ND and −ND suspensions. A suggested mechanism for these observations is that carboxylated −NDs from aqueous suspensions are forming robust lubricious deposits on stainless and alumina surfaces that enable gliding of the surfaces through the −ND suspensions with relatively low resistance to shear. In contrast, +ND suspensions are failing to improve tribological performance for either of the surfaces and may have abraded existing protective boundary layers in the case of stainless steel contacts. This study therefore reveals atomic scale details associated with systems that exhibit starkly different macroscale tribological properties, enabling future efforts to predict and design complex lubricant interfaces.

## Introduction

**Figure 1:**
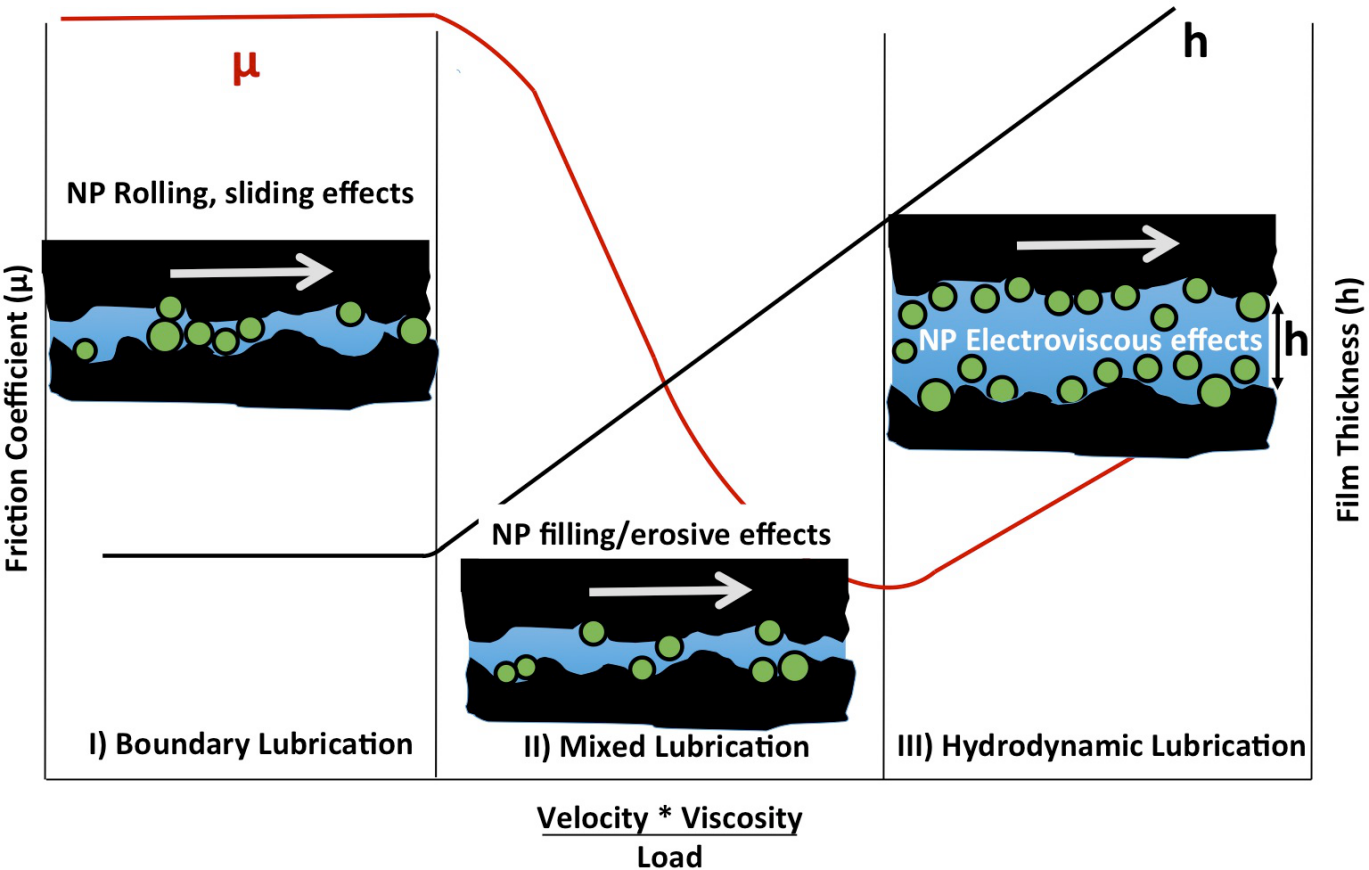
Friction coefficient plotted as a function of fluid viscosity and shear velocity divided by load (Stribeck curve). NPs present in a fluid could impact the tribological performance in all three lubrication regimes, for example by forming lubricious surface coatings or acting as rolling or sliding spacers at the contacts. In addition they may potentially fill or erode the contacts and/or cause the solid–fluid interfacial slip attributes to change via electroviscous and/or steric mechanisms. See text for further discussion.

Interest in nanoparticles as eco-friendly lubricant additives has grown tremendously in recent years [1–2]. The field is driven in a large part by a pressing need to replace hazardous additive materials in present-day oil-based lubrication technologies and to eliminate the serious environmental risks associated with oil leakage and disposal [3–5]. Water-based lubricant systems are a particularly attractive target for nanoparticulate additives since conventional oil additives generally fail to improve tribological performance in aqueous environments. Numerous studies of nanoparticulate additives to oil-based systems have been reported in the literature, with many displaying significant improvements in macroscopic friction and wear rates [6]. Water-based suspensions have received far less attention [1–2,7–9]. Although the low shear strength of water is beneficial in the hydrodynamic regime of lubrication, under normal loads it also enables contact between opposing surfaces. Nanoparticulate additives have the potential to overcome this deficiency, by penetrating into contacts where they may form boundary films and/or act as rolling or sliding spacers (Figure 1) [6,10–11]. As such, nanoparticles exhibit a great potential for replacement of the centuries-old oil-based lubricating technologies.

Tribological studies of water-based nanoparticle suspensions reported to date have mostly involved NDs. Reductions in kinetic friction coefficients µ_k_ by factors of 5–20 have been reported for metallic [7], ceramic [8], and semiconducting materials [2]. It is likely that the literature is reporting primarily on those nanoparticle additives with a beneficial tribological performance. Identification of nanoparticulate additives that are detrimental and/or have no effect have received much less attention even though such data are exceptionally useful for the purposes of evaluating test models [12]. Liu et al. recently investigated the tribological performance of steel/gold contacts in water using both nano- and macroscale measurements and found the contact to be highly sensitive to the sign of the charge on the NDs in suspension [9]. The authors suggested that the −ND suspensions were more likely to improve the tribological performance in macroscale settings than the +ND suspensions, and speculated that the electrostatic properties of the materials in contact might play a role. Generally, NDs require surface chemical treatments in order to be electrically charged in aqueous suspensions so as to inhibit aggregation via a mutual electrostatic repulsion, similar to other nanoparticles [2,9,13–15]. These chemical treatments are well known, however, to impact the friction coefficients in humid and dry environments for standard tip on disk geometries [16–17]. The surface chemical treatments employed in the production of the ND might therefore dominate the tribological performance. The surface charges on ND are also expected to affect the interfacial solid–fluid slip lengths attributes, and therefore the apparent fluid viscosity, via electroviscous and/or steric mechanisms [18–20]. Fundamental studies at the nanoscale are clearly essential at this time in order for the field to progress and for accurate model predictions to be developed.

QCM is emerging as an ideal tool for studying the fundamental mechanisms associated with nanoparticle lubrication [9]. While historically it was developed as a time standard and a deposition rate monitor for thin films [21], it has rapidly expanded in recent years to a broad range of applications through simultaneously monitoring of changes in frequency and quality factors [22–26]. It has become well known as a nanotribological technique for studying uptake and sliding friction levels of films in both in vacuum and liquid environments [23–25]. When immersed in liquid, it can be used to probe frictional drag forces and interfacial effects at complex solid–liquid interfaces [19,27] including those of a biological origin [18,22]. Given that the transverse shear speed of the oscillating QCM electrode is generally in the range of mm/s to m/s [23], it can readily be compared to conditions of macroscopic friction measurements. In addition, QCM experiments can be performed using an electrode in a rubbing contact with another macroscopic surface, for example a ball bearing [9,26], yielding important information on the shear strength and friction coefficients associated with macroscopic contacts.

**Figure 2:**
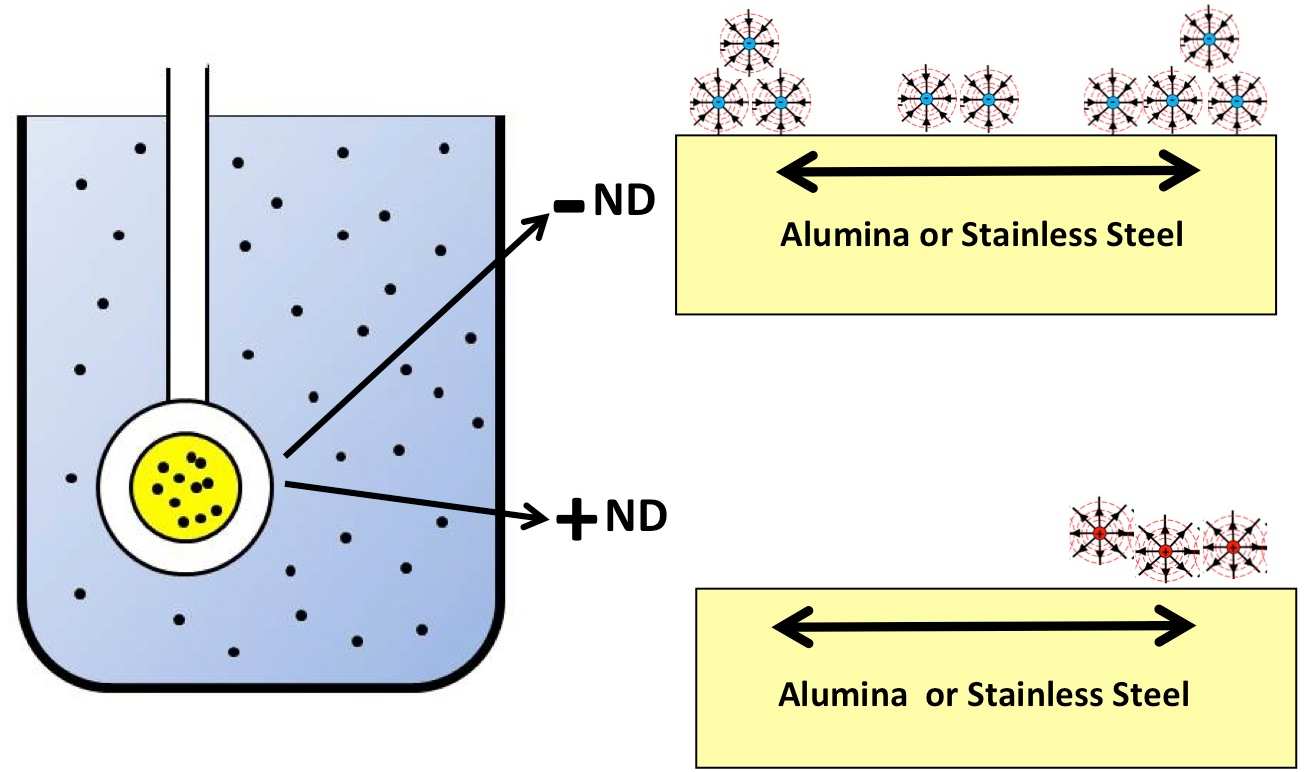
Schematic of a QCM immersed in aqueous suspensions of −ND and +ND, for sliding friction studies on materials with contrasting electrical properties, namely alumina and stainless steel. Adapted with permission from [9], copyright 2015, Royal Society of Chemistry.

For the present study, the QCM technique was employed to perform a comparative analysis of the tribological parameters of aqueous suspensions of either positively (hydroxylated) or negatively charged (carboxylated) NDs for the surfaces with contrasting electrical properties, namely insulating ceramic (alumina) and electrically conducting (stainless steel) surfaces immersed in suspensions of either positively (hydroxylated) or negatively charged (carboxylated) NDs (Figure 2).

The QCM measurement experiments were complemented by AFM and SEM measurements of the surface topography before and after the ND exposure, as well as macroscale measurements of µ_k_. The materials were inspired by Liu et al.’s suggestion that differences in the tribological properties between +ND and −ND suspensions might originate in electrostatic effects [9], since the electrical charge carriers in the QCM electrodes might respond differently to positively and negatively charged nanoparticles. Would the effect therefore be absent for insulating materials? Was the explanation viable given the symmetry of electrostatic forces?

As will be reported, beneficial tribological behaviors were observed for immersion of stainless steel or alumina samples in −ND suspensions, while either neutral (alumina) or detrimental (stainless steel) behaviors were observed for immersion in +ND suspensions. This yields an exceptional opportunity for cross-comparisons with atomic scale tribological probes. At the atomic scale, the QCM and microscopy studies indicated uptake of particles, along with the potential presence of lubricious slurry for the −ND suspensions, somewhat analogous to boundary lubrication and steric repulsion effects by mucinous glycoproteins boundary layers in aqueous biological settings [18]. Such behavior was not observed for the +ND suspensions. The nanoscale mechanisms associated with effective lubrication therefore include boundary film deposits in combination with low interfacial resistance to shear motion in the suspension.

## Materials and Methods

### Materials

Aqueous suspensions of 5 nm detonation NDs with oppositely charged zeta potentials were purchased from Adamas Nanotechnologies (Raleigh, NC). All other chemicals were purchased from Sigma-Aldrich (St. Louis, MO) or Acros Organics (Morris Plains, NJ). The −ND samples were carboxylated [2,28], (part# ND5nmNH20) and as manufactured have an average particle size of 5 nm and a zeta potential of −50 mV [29]. The +ND samples were hydroxylated in the course of a reduction reaction [28], (part# ND5nmPH20) and, as manufactured, have an average particle size of 5 nm and a zeta potential of +45 mV. The suspensions were employed as received from the manufacturer in the form of 1 wt % slurries in DI water, and stored without exposure to light. The suspensions were diluted tenfold by volume in advance of experiments using DI water to yield 0.1 wt % suspensions employed in all measurements. While both stock and the diluted suspensions were found to be stable over a short storage up to 1 month, some slow agglomeration has been observed over a prolonged storage (e.g., see [9,29]).

Macroscale friction measurements were performed with ball-on-disk contacts of like materials. Alumina (Al_2_O_3_) ball and the disk contacts were purchased from PCS Instruments (London, United Kingdom), with respective part #’s MTMB3/4AL2O3 and MTMD3/4AL2O3.

Stainless steel (AISI 52100) polished ball and the disk contacts were also purchased from PCS Instruments (London, United Kingdom), with respective part #’s BALLD and MTMPD. Unpolished 304 stainless steel penny washers (part # HYW-M10-45-A2) were purchased from AccuGroup, (Huddersfield, United Kingdom) (304 stainless steel is also referred to as A2 stainless steel) and were employed as disks for selected measurements. Before the macroscale friction measurements all surfaces were cleaned in ethanol and then DI water.

5 MHz polished QCM crystals (1” diameter) with either stainless steel (SS304; part number QM1022) or aluminium (part number QM1010) electrodes on the liquid facing side were purchased from Fil-Tech (Boston, MA). The QCM’s were specifically designed for operating with one surface immersed in a liquid at a fundamental transverse shear mode. The aluminum QCM samples were anodized using a literature method that grows an alumina layer at the rate of 2 μm/h [30]. For these results, the liquid side QCM electrode was connected as the anode and the sample was then immersed into 4 wt % oxalic acid solution maintained at 0 °C. A cathode was placed in the bath and an electric potential of 40 V was applied between the anode and the cathode. Anodization was halted at 3 min yielding an approximately 100 nm thick Al_2_O_3_ layer. After the anodization procedure, the samples were thoroughly rinsed with DI water before mounting the sample within the flow cell.

### Macroscale friction measurements

Macroscopic scale friction coefficient measurements were performed with a MTM2 Mini-Traction Machine (PCS Instruments, London, UK). The apparatus is capable of measuring frictional properties of both lubricated and unlubricated contacts under either both sliding and rolling conditions. Its test specimens consist of a 19.05 mm (3/4 inch) ball and a 46 mm diameter disc. The ball is loaded against the face of the disc and the ball and disk are driven independently to create a mixed rolling/sliding contact. Force transducers measure the frictional forces, and additional sensors are present to measure the loading force and lubricant temperature in real time. For the measurements reported here, the setting were adjusted to a normal load of 4 N with a ball rolling speed of 200 mm/s and a slide to roll ratio of 90%, which resulted in a smooth friction coefficient versus time signal for both stainless steel and alumina contacts. Lubrication under such conditions allows one to probe of the ability of nanoparticulates to penetrate the contacts when they are introduced to fluids surrounding a contact [10].

A series of alumina–alumina and stainless steel ball on disk combinations were studied, ranging from pristine as-manufactured samples to samples that had experienced multiple exposures to DI water and ND suspensions. Contacting materials included both the alumina and stainless steel substrates obtained directly from the instrument manufacturer or stainless steel penny washers situated in place of the disk while in contact with the stainless steel ball.

### Atomic force microscopy and scanning electron microscopy characterization of surface topology

Surface topology characterization of the QCM electrodes was performed with an Asylum Research MFP 3D AFM equipped with silicon nitride tips (part#NCHV-A, Bruker AFM Probes, Camarillo, CA) and operated in a tapping mode. The 1024 × 1024 images were recorded at a rate of 1 line/s yielding a height profile *h* = *h*(*x*_i_,*y*_i_). The height profiles were quantified by the rms roughness value σ, which is virtually always dependent on the size of the area sampled below a characteristic lateral correlation length ξ. For a self-affine fractal surface the rms roughness increases with the lateral length of the sampled area as σ 
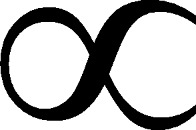
 *L^H^*, where *H* is the roughness exponent whose value lies between 0 and 1. Fractal surfaces are often characterized by self-affine fractal dimension *D* = 3 − *H* [31–33]. Self-affine surfaces have an upper horizontal cut-off length (the lateral correlation length (ξ)) above which the rms roughness saturates towards a value of σ_s_ and no longer exhibits fractal scaling. The surface roughness parameters (D, ξ and σ_s_) reported herein were obtained from the log(σ) vs log(scan size) plot method as described by Krim and co-workers [32]. Previously, a detailed comparison of the results obtained by this method to several literature approaches yielded roughness parameters within experimental error of each other [33]).

Scanning electron microscope imaging was performed using an FEI Verios 460L field-emission microscope. A high resolution through-the-lens detector was used in a beam-deceleration mode for ultra-high resolution backscatter imaging of flat samples. For a typical SEM imaging, a whole QCM crystal (1” in diameter) was attached to a pin mount using a small piece of a double sided carbon tape. Sets of images at different magnifications ranging from 5 × 10^3^ to 350 × 10^3^ were recorded under typical settings of an accelerating voltage and a bias of 2.00 kV and 200 V, respectively.

### Quartz crystal microbalance apparatus

QCM data were collected using a QCM100 (Stanford Research Systems, Sunnyvale, CA, USA) system. The system includes a controller, oscillator electronics and a Teflon holder and a flow cell that exposes one side of the crystal to approximately 0.15 mL of liquid, as well as providing mechanical support and electrical connections to the QCM electrode. All QCM experiments were carried out at room temperature and the temperature was stabilized by the thick, insulating polymer walls of the flow-cell apparatus, as evidenced by the flatness in the frequency during initial and final water exposures. All liquids were kept adjacent to one another on the lab bench during the experiment to minimize temperature differences between them. A LabView (National Instruments, Austin, TX) PC-based data acquisition system was used to record both the crystal resonant frequency and the conductance voltage *V*_c_, from the controller output. The conductance voltage, *V*_c_, is related to the mechanical resistance, *R*_m_*_,_* as 
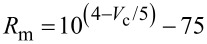
 [34]. Changes in mechanical resistance are directly proportional to changes in the inverse quality factor of the resonator, as will be described below.

Data were recorded as follows. The QCM sample was first placed into the flow-cell initially filled with ambient air. The system was operated continuously until the frequency and amplitude stabilized to less than 1 ppm/min. Once this stabilization occurred, DI water was injected into the flow-cell. Following 1 h water exposure, 10 mL of aqueous 0.1 wt % ND suspension was flushed into the flow-cell using a syringe pump. The use of a large excess of ND suspension ensured complete replacement of the DI water in the cell. Following 1 h of exposure to the ND suspension, 20 mL of DI water was flushed into the cell by a second syringe pump.

### Quartz crystal microbalance data analysis

Analogous to the description in [9], changes in the resonant frequency, δ*f* , and the inverse quality factor, δ(*Q*^−1^), of a QCM reflect changes in the mass and frictional energy losses of materials deposited onto its surface electrodes and/or drag forces and interfacial slippage of fluids that it is immersed in. For a QCM with one side immersed in a fluid with bulk density ρ_3_ and viscosity η_3_, the shifts in δ*f* and δ(*Q*^−1^) associated with the presence of the liquid under no-slip boundary conditions are given by [35]:

[1]
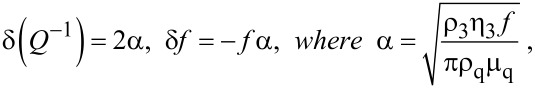


where ρ_q_ = 2.648 g/cm^3^ is the density and µ_q_ = 2.947 × 10^11^ g/cm/s^2^ is the shear modulus of quartz. Immersion of one side of a 5 MHz resonant frequency QCM in water at room temperature (ρ_3_ = 1 g/cm^3^, η_3_ = 0.01 poise) results in a δ*f* = −714 Hz drop in the resonant frequency and an increase of δ(*Q*^−1^) = 2.85 × 10^−4^ in the dissipation. For a QCM with quality factor *Q* = 50,000 in air this corresponds to a drop to *Q* = 3,280 after an immersion in water.

The viscous drag forces on the QCM electrode are mechanical in nature; a decrease in *Q* is manifested as an increase in the series resonant resistance *R*_m_ of the QCM resonator that can be measured electrically. For a QCM electrode exposed to a fluid from one side under non-slip conditions [36–37]:

[2]
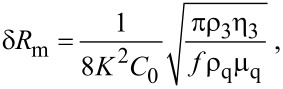


where *K*^2^ = 7.74 × 10^−3^ is the electromechanical coupling factor for the AT cut quartz (AT stands for temperature compensated transverse shear mode type A) and *C*_0_ is the static capacitance of the QCM electrodes, including the parasitic capacitance associated with the connections to the oscillator circuit. A comparison of Equation 2 and Equation 3 reveals that δ*R*_m_ is directly proportional to δ(*Q*^−1^): Both are reflective of the oscillator dissipative behaviour. For the QCM system employed here, the theoretical value for δ*R*_m_ increase associated with the immersion of a 5 MHz resonant frequency QCM in water is approximately 300 Ω.

In practice, the frequency shifts observed upon immersion into a liquid environment are larger than those predicted by Equation 1 for perfectly planar QCM electrodes. This effect is attributed to the roughness of the surface electrode and can be minimized by using overtone polished crystals, but cannot be completely neglected because no surface has perfectly zero roughness. The magnitude of this contribution has been estimated to be in the range of 2–10% for materials with rms roughness of the same order as the QCM electrodes employed here [36–41]. Therefore, if a QCM surface immersed in a liquid becomes rougher or smoother while immersed in a liquid suspension of nanoparticles, its frequency will drop or increase in unison. A similar response is present also for the changes in the mechanical resistance.

In addition to the aforementioned contributions, nanoparticles may rigidly adhere to the surface when introduced into a liquid, resulting in further changes to the frequency and quality factor of the QCM in association with their mass loading effects. As originally reported by Sauerbrey, an additional rigidly adhering film deposited onto one side of a QCM will decrease its resonant frequency by [21]:

[3]
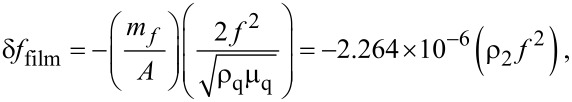


where ρ_2_ = (*m_f_*/*A*) is the mass per unit area of the film in g/cm^2^. This equation is the main basis for the use of QCM as a mass sensor in vacuum applications where the Equation 1 contributions from a surrounding fluid are absent. Martin et al. studied the effect of simultaneous mass and liquid loading on QCM and demonstrated that Equation 1 and Equation 3 can be added linearly to obtain the combined effect so long as the mass is not slipping on the surface electrode [36]. Therefore the frequency shift associated with mass uptake from a liquid is the same as mass uptake from a vacuum.

If the adsorbed particles slip on the QCM surface in a response to the oscillatory motion, and/or the no-slip boundary conditions are altered at the upper boundary of the film with the surrounding liquid, the magnitude of the frequency shift δ*f*_film_ will be lower than that of a rigidly attached film [27,37,40]. These effects may cause the liquid’s effective viscosity to appear to increase or decrease due to electroviscous or steric effects [19]. They will also be reflected in the QCM’s quality factor, *Q*, since the friction associated with the oscillatory motion is manifested in the quality factor. Therefore, while the exact details of the complex solid–liquid–nanoparticle interface may be unknown, changes in the quality factor reflect outright the frictional resistance forces at the interface, and in particular whether the combined resistance to shear motion at the solid–liquid interface. Frequency shifts due to changes in temperature and/or stress on crystal by the added mass layer are expected to be very minimal for the present work, since the measurements were performed at constant room temperature.

## Results

### Macroscale friction measurements

**Figure 3:**
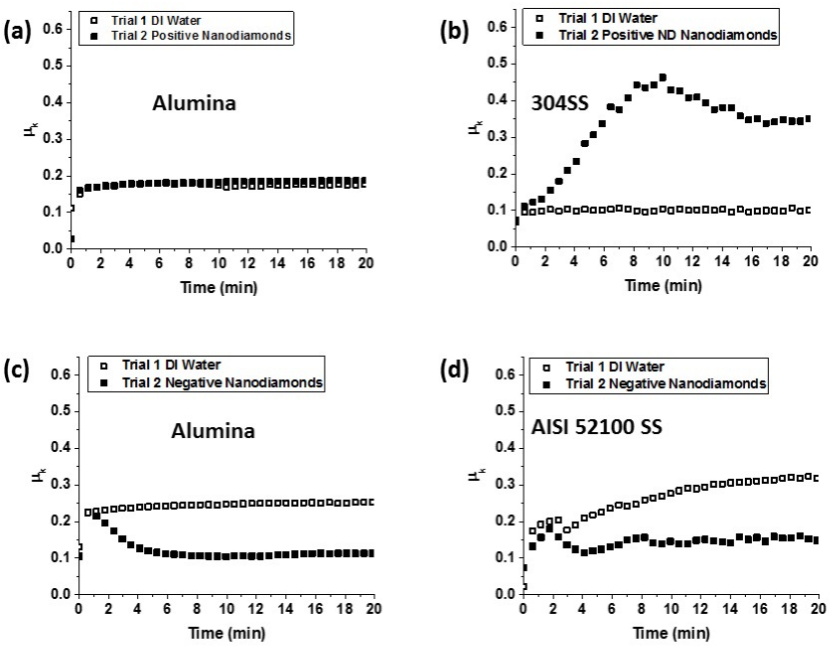
Representative friction coefficient versus time plots for alumina (left) and stainless steel (right) contacts lubricated with pure DI water (open squares), positively (a,b) and negatively (c,d) (filled squares) charged ND suspensions. Addition of −ND to water (c,d) consistently lowered the friction coefficient while addition of +ND to water consistently (a,b) failed to lower the friction coefficient.

Figure 3 shows representative data recorded in a macroscale friction experiment when the contacting surfaces become exposed to ND suspensions. The data reveal a clear difference between the surfaces exposed to +ND and −ND suspensions. Introduction of −ND suspensions consistently resulted in a substantial reductions in µ_k,_ to the range of 0.05–0.1 while +ND consistently resulted in a modest to substantial increases in µ_k_. Substantial increases in µ_k_ were observed for the stainless steel surfaces exposed to the +ND suspensions while alumina surfaces showed only modest to negligible increases in µ_k_. The data do not appear to correlate with the σ_s_ or fractal dimension *D* of the samples [42], which were measured by AFM to respectively be (6 nm, 2.4); (9 nm, 2.2); and (50 nm, 2.1) for the polished AISI 52100 stainless steel disk, 304 stainless steel penny washer, and alumina disk samples.

After the experiments with ND suspensions, selected samples were removed, cleaned extensively in DI water in an ultrasonic bath, and then measured again. In these trials, µ_k_ remained unchanged for samples that had been exposed to +ND suspensions, and increased slightly for those which had been exposed to −ND suspensions. Exposure of the samples to a ND suspension of the opposite sign would, however, immediately alter the friction coefficient. For example, if a sample was immersed in a ND suspension with an opposite surface charge to its first exposure, µ_k_ would shift up or down depending on the sign of the ND charge in the second exposure.

The observations suggest that −ND have a strong affinity for the surfaces studied and act as passivation agents. They also reveal that −ND and +ND may act as neutralizing or removal agents for one another. In order to probe ND attachment/film formation and/or polishing effects for the surfaces as a whole and to separate this from effects confined within the contact region, AFM and QCM measurements were performed on surfaces exposed to ND suspensions in the absence of a contacting load. In the absence of a contact, the ND are still potentially abrasive, on account of the oscillatory nature of the QCM electrode and the associated high acceleration rates.

### AFM and SEM measurements

AFM measurements of open surfaces exposed to ND suspensions were performed directly on samples employed for the QCM studies so as to be able to directly cross-reference results obtained from the two techniques. Images were recorded in air, after 1 h of QCM oscillation while immersed in water and after one hour of QCM oscillation while immersed in a ND suspension. The high frequency nature of the oscillation in the presence of the NDs slurries could potentially remove the electrode material but NDs might also attach to the surface. AFM measurements were recorded in at least triplicate for each unique solid:ND combination.

**Figure 4:**
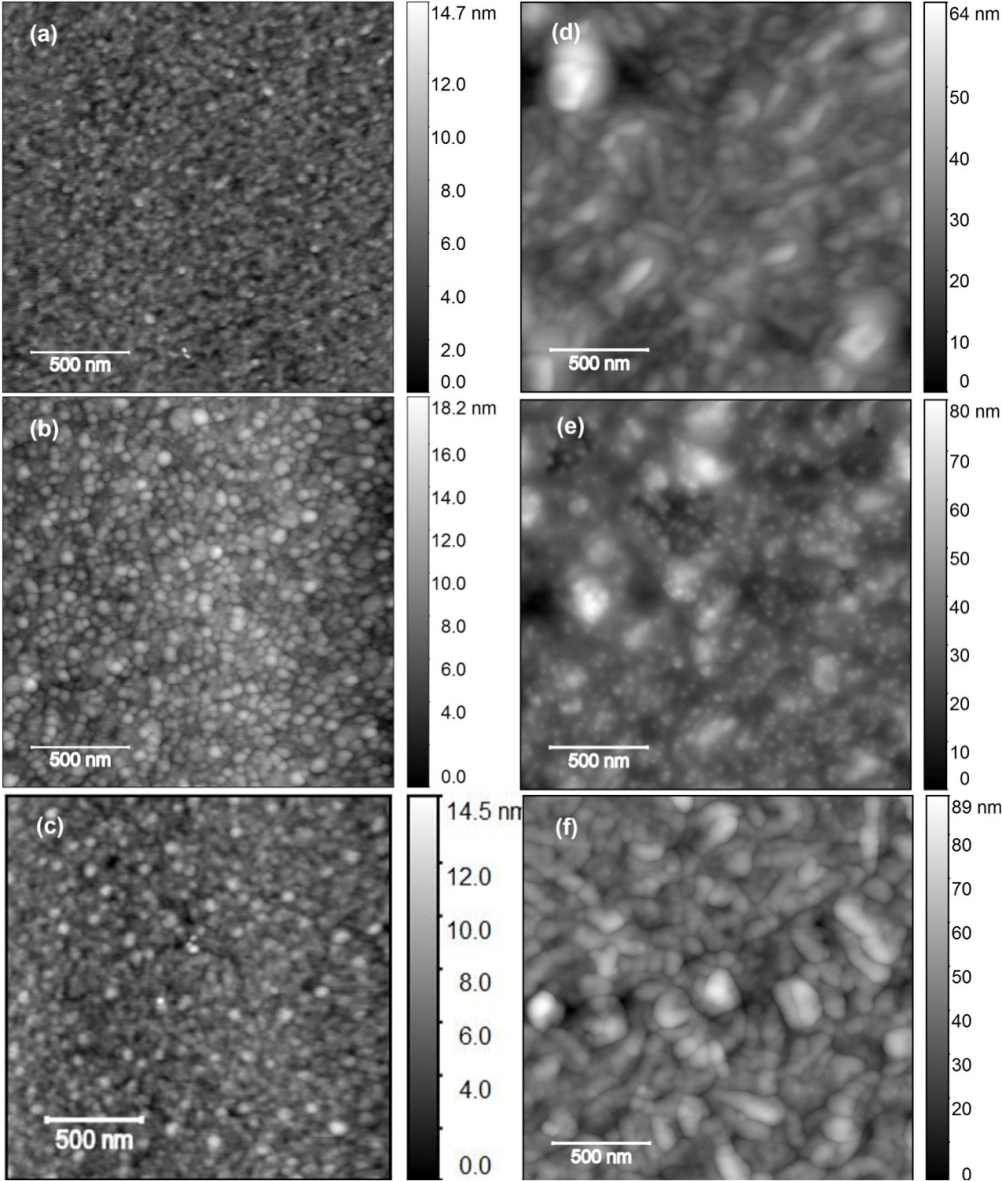
Representative AFM images of stainless steel 304 (left) and alumina (right) QCM electrodes after 1 h of oscillation in DI water (upper), −ND (middle) and +ND (lower) suspensions and then rinsing in DI water.

Figure 4 shows representative images of stainless steel 304 (left) and alumina (right) QCM electrodes after oscillating in DI water for 1 h, −ND and +ND suspensions for 1 h , and then rinsed in DI water. Images (b), (c) and (e) for alumina and SS304 surfaces immersed in −ND and SS304 surfaces immersed in +ND indicate changes to the surface electrodes, with (e) the alumina surfaces exposed to −ND exhibiting the most pronounced differences as compared to the water exposure only. Image (f) for alumina surfaces exposed to +ND meanwhile shows no visual evidence of NDs. The Figure 4 images do not specifically reveal whether substrate material was removed in a polishing process during immersion as has been clearly documented for gold QCM electrodes in aqueous suspensions of SiO_2_ [43]. While alumina and SS304 may not be as susceptible to erosion and polishing as gold, NDs could potentially remove the electrode material by an accelerated penetration into surface asperities by the oscillatory action of the QCM [44–46].

**Figure 5:**
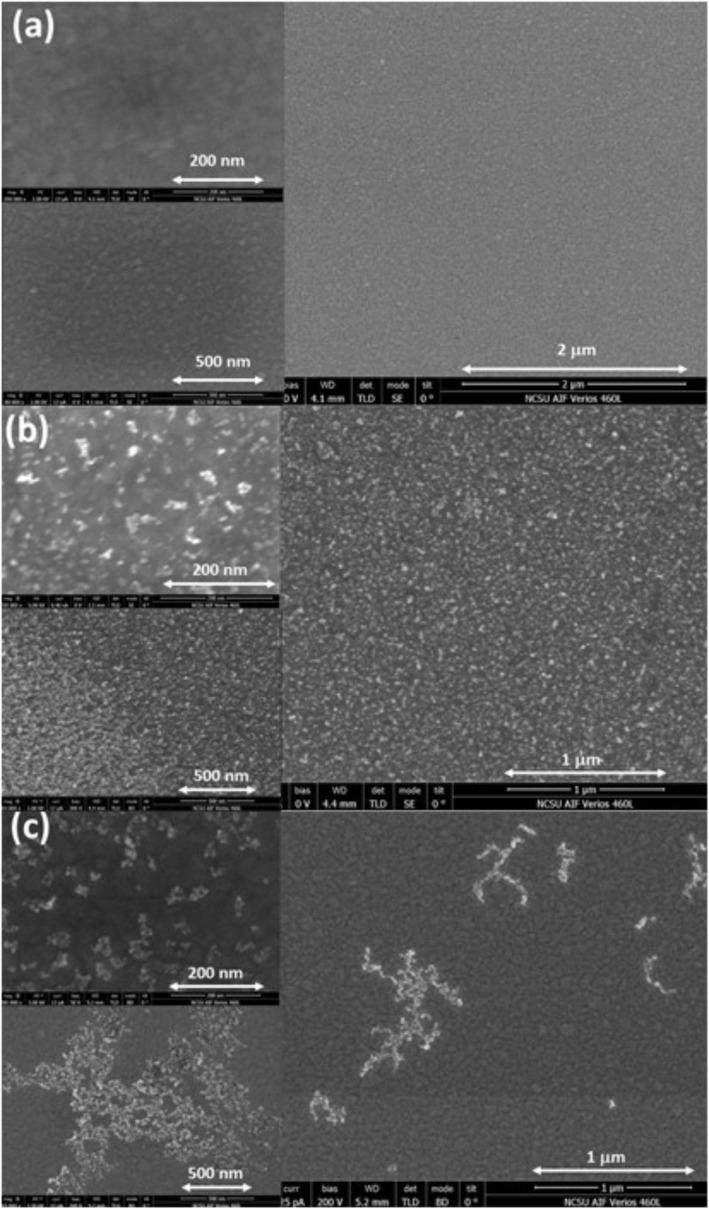
SEM images of SS304 QCM electrodes after oscillated in (a) water, (b) −ND, and (c) +ND suspensions for 1 h and then rinsed in DI water. See text for further details.

Additional scanning electron microscopy (SEM) images (Figure 5) were recorded on the SS304 samples to further elucidate changes in the surface topology after exposure to NDs followed by rinsing in DI water. Alumina samples became rapidly charged upon an exposure to an electron beam (presumably due electrically isolating alumina layer still present on the electrode surface), preventing high quality SEM images from being recorded. Features associated with permanently adhering nanoparticles are present in the images for the samples exposed to ND. For the case of −ND exposure, the attached particles are clustered and the deposits are more uniformly distributed over the surface. The +ND exposure results in very sparse deposits, which form dendritic surface aggregates in regions where they are present.

The AFM and SEM data are consistent with the results from nanodiamond seeding literature [46], where it has been reported that the particle attachment density can, for example, vary from very low (10^8^ cm^−2^) for hydrogen treated nanodiamonds (+ND) to very high (10^11^ cm^−2^) for oxidized nanodiamonds (−ND) on AlN substrates [47]. The data are also consistent with a report on a formation of ND clusters of ca. 23 nm in diameter on SiO_2_ surfaces exposed to ND dispersions [48]. Clusters of this size are large enough to separate the surfaces employed for the Section ‘Macroscale friction measurements’.

In order to characterize adhesion of NDs to surfaces quantitatively, the QCM frequencies and resistances were compared for crystals exposed to air before and after the exposure to the ND suspensions (followed by a rinse in pure water). The results, summarized in Table 1, exhibit a decrease in frequency after the exposure to the suspensions regardless of the ND surface charge sign. While some of the downward shifts in frequency may be attributable to a variation in the uptake of particulate and physisorbed species from air, the net mass increase is consistent with the addition of NDs in levels that exceed the mass of any material removed from the QCM electrode arising from the potentially erosive action of the ND. Consistent with the images, a definitive mass uptake is present for the alumina sample immersed in the −ND suspension. Resistance shifts were virtually zero for the stainless steel samples and consistent with the rigidly attached NDs. The increase in resistance for the alumina samples immersed in +ND is consistent with the presence of poorly attached layers that are slipping on the surface. This might be attributable to loosely attached NDs or physisorbed adsorbates, neither of which would be readily observed by AFM.

**Table 1:** QCM frequency (+/−15 Hz) and resistance shifts (+/−1 Ω) in air before and after 60 min of oscillation in aqueous ND suspensions. The equivalent surface coverage of 25 nm clusters is also reported (+/−1 × 10^10^ clusters/cm^2^).

Sample	+ND suspension immersion		25 nm cluster coverage level	−ND suspension immersion		25 nm cluster coverage level

	δ*f* (Hz)	d*R* (Ω)	clusters/cm^2^	δ*f* (Hz)	d*R* (Ω)	clusters/cm^2^
alumina	−8	+15.5	0.7 × 10^10^	−69	−1.6	6.1 × 10^10^
SS304	−18	−0.1	1.6 × 10^10^	−25	−0.1	2.2 × 10^10^

In order to convert the frequency shifts to particle density on the surface, we assume a cluster size of 25 nm and a packing fraction within the cluster of 0.7. The mass of each cluster would be [(25 nm/5 nm)^3^] × 0.7 (2.29 ×10^−19^ g/5 nm particle) = 2 × 10^−17^ g. A surface coverage of 10^10^ clusters per cm^2^ therefore has a mass per unit area of ρ_2_ = 2 × 10^−7^ g/cm^2^, which corresponds to a decrease in the resonant frequency of 11.3 Hz (cf. Equation 3). For comparison, a monolayer of spherical 5 nm diamond nanoparticles packed in the closest hexagonal arrangement (assuming diamond bulk density of 3.5 g/cm^3^; mass per particle: 2.29 × 10^−19^ g) corresponds to 4.6 × 10^12^ ND/cm^2^, ρ_2_ = 1.058 × 10^−6^ g/cm^2^ and a decrease in the resonant frequency of 59.8 Hz.

**Figure 6:**
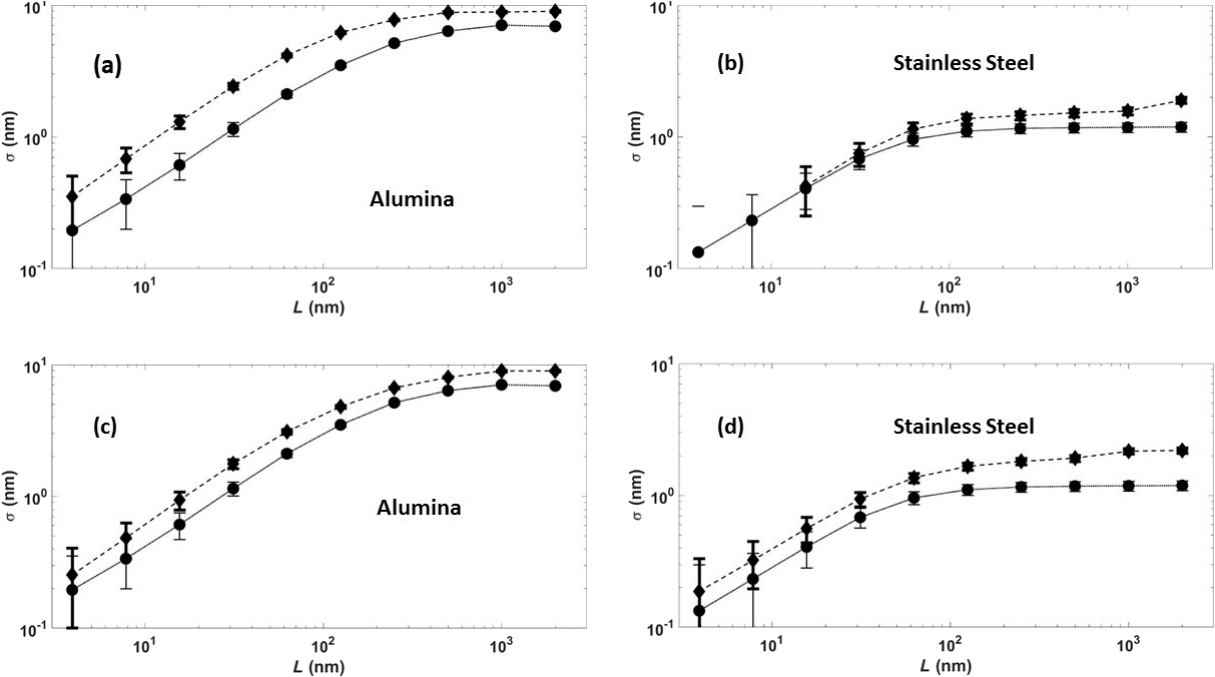
RMS roughness σ versus scan size *L* for QCM electrodes comprised of alumina (left) and stainless steel (right) after an oscillation for 1 h in DI water (solid lines) and after an oscillation for 1 h in suspensions of either positively (a),(b) or negatively (c),(d) (dashed lines) charged NDs. See text for details.

Graphs of log(σ) vs log(scan size) obtained from the AFM images shown in Figure 4 are presented in Figure 6. Each data point represents an average of multiple locations on the surface. The slope of a linear fit in lower length scale gives the roughness exponent (*H*), and an exponential fit for the larger length scale gives the asymptotic value of σ_s_, as described earlier.

All samples exhibited increases in σ_s_ after an oscillation in ND suspensions (Table 2). Alumina surfaces, however, exhibited greater increases in σ_s_ than SS304. Only the SS304 sample exposed to +ND exhibited a change in *D*, increasing from 2.2 to 2.3, which corresponds to a more jagged surface texture [30]. It is interesting to note that this is the only surface studied that exhibited a striking increase in friction upon an exposure to the NDs.

**Table 2:** Saturated rms roughness σ_s_ (+/−0.1 nm) and fractal dimension *D* (+/−0.05) of QCM electrodes after 1 h of oscillation in DI water or ND suspensions.

Samples	Pure DI water		+ND suspension		−ND suspension	
	σ_s_ (nm)	*D*	σ_s_ (nm)	*D*	σ_s_ (nm)	*D*

alumina	7.8	2.1	9.8	2.1	9.9	2.1
SS304	1.2	2.2	1.6	2.3	2.4	2.2

### QCM measurements

**Figure 7:**
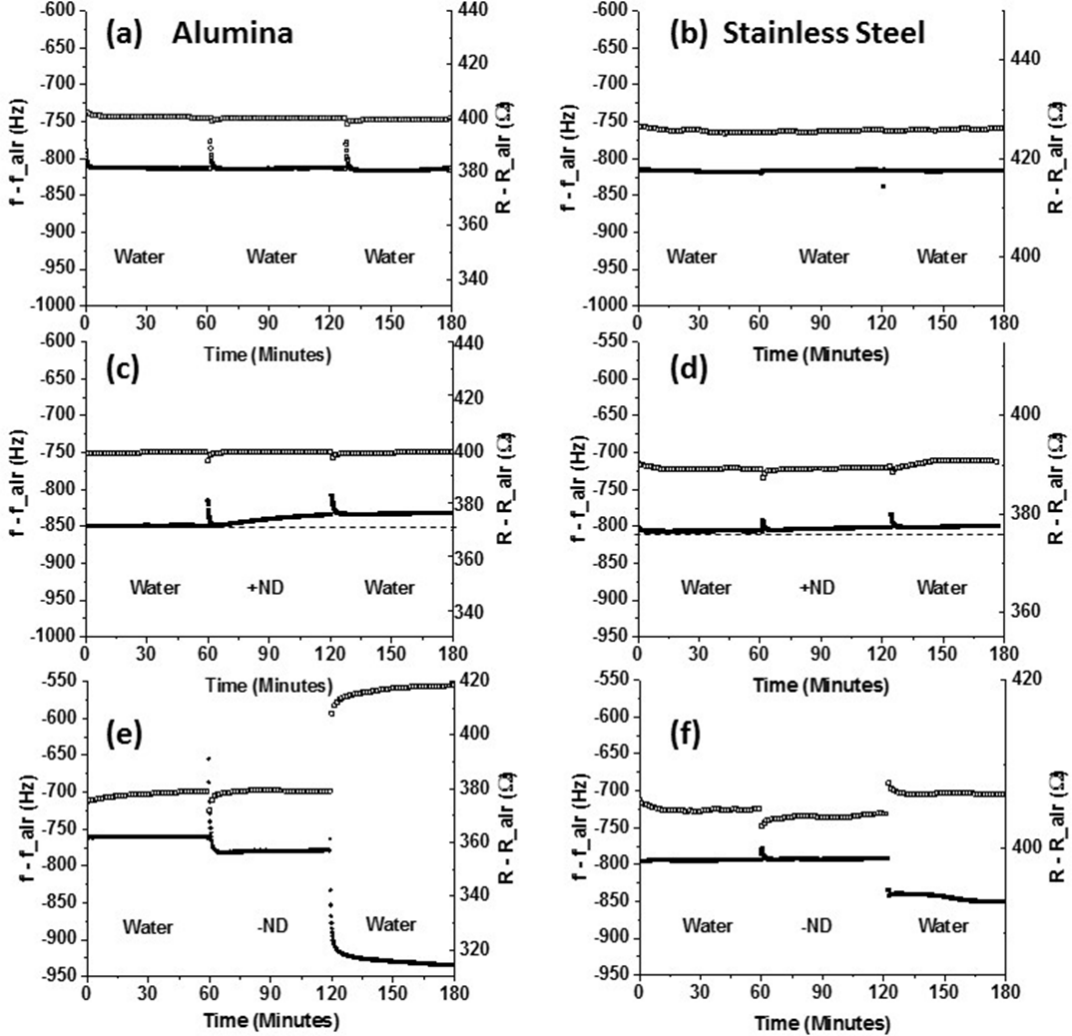
Time course of changes in mechanical resistance, *R* (top, open squares), and frequency *f* (bottom, filled squares), of QCM relative to air for SS304 (right) or alumna (left) electrode surfaces consequently exposed to DI water (a and b); +ND and water (c and d); −ND and water (e and f).

Frequency *f* and mechanical resistance *R* values of QCM relative to their initial values in air, *f*_air and *R*_air, are summarized in Figure 7. All QCM crystals were first exposed in DI water for 1 h followed by +ND or −ND dispersions for another 1 h and then returned to DI water for an additional 1 h. Fluid injections at 1 and 2 h in some cases caused temporary perturbations in *f* and *R* that serve as markers delineating the three regimes of exposure. All experiments were conducted at in at least triplicate using new QCM crystals, and only minor differences were observed between the individual runs.

Control runs in DI water (i.e., no ND exposure) are displayed in Figure 7a and 7b for the alumina and SS304 electrodes. Only minimal changes in *f* and *R* are observed in the control runs, apart from the momentary perturbations occurring at the time of pure water injections. Also, the drops in *f* and *R* relative to those recorded air are larger than the theoretical values for perfectly planar surfaces, −714 Hz and 300 Ω. These large experimental shifts are attributable to the surface roughness of the electrodes.

Changes in *f* and *R* for the samples exposed to ND suspensions were found to be dependent on the ND surface charge. Both the alumina and SS304 samples exhibit a slow, yet a small increase in *f* upon an exposure to +ND suspension (Figure 7c and 7d) that ceases upon re-entry of DI water. Given the AFM results indicating that both surfaces become slightly rougher upon an exposure to +ND, the upward trend does not appear to be attributable to the surface polishing but rather some modest erosive effects. Virtually no changes in the resistance to shear motion at the solid liquid interface were observed upon an introduction of the +ND. Such changes might result from loosely bound particles enabling some decoupling of the mass of the fluid surrounding the QCM with no significant reductions in the friction energy losses at the interface.

In contrast, significant changes in both *f* and *R* observed upon an exposure of both types of surfaces to −NDs and consequent rinsing in DI water (Figure 7e and 7f). Specifically, upon exposing the QCM to −ND suspension both *f* and *R* abruptly drop for the alumina sample while for the SS304 sample the frequency is essentially unchanged while *R* drops abruptly. Upon rinsing, an abrupt drop in *f* and a rise in *R* are observed for both surfaces. The QCM data provide a clear evidence of the −ND exposure permanently altering the both surfaces. These surface alterations appear to have a direct effect on the shear forces encountered at the solid–liquid interfaces. This global surface treatment may in fact provide a key piece of evidence to the fundamental mechanism underlying the friction reductions observed at the macroscale for the stainless steel and alumina surfaces immersed in −ND suspensions.

## Discussion

The data reported here reveal key similarities and differences in both the macro- and nanotribological properties of stainless steel and alumina when exposed to ND aqueous dispersions. While Liu et al.’s suggestion that −ND dispersions are more likely to improve the tribological performance at the macroscale than the +ND dispersions [9] has been validated, the previous explanation of the underlying mechanism by strictly electrostatic appears to be somewhat simplistic within the context of simply comparing insulating alumina and conducting stainless steel surfaces.

For QCM electrodes coated with aluminum, the surface-exposed aluminum metal is readily oxidized to Al_2_O_3_ under ambient air. This surface layer of alumina is protecting the rest of metal from a further corrosion. In our experiments, the electrodes were additionally anodized for 3 min. This procedure is expected to yield approximately 50–100 nm thick Al_2_O_3_ layer. For Al_2_O_3_ is exposed to water one expect a formation of several aluminum hydroxide phases [49]. The presence of hydroxy (OH) groups on the alumina surface is affected by the oxide surface structure as well as other parameters with pH being the most important. Overall, the surface hydroxy groups determine electrostatic properties of the surface that, in turn, affect interactions of charged nanoparticles (NDs in this study) with the QCM alumina electrode.

In the past Cuddy et al. employed contact angle titration to determine Isoelectric points (IEPs) for five common (QCM) sensors [50]. Specifically, they reported a mildly basic IEP = 8.7 for Al_2_O_3_ sensors. Therefore, the QCM alumina surface is likely to be positively charged at the neutral pH of our experiments and this would explain a rapid uptake of negatively charged NDs on the electrode surface observed in our QCM experiments. Thus, this observation is in agreement with Liu’s electrostatic hypothesis [9]. We note that recently an electrostatic self-assembly seeding of monosized individual diamond nanoparticles (obtained by a detonation method) on silicon dioxide surfaces has been reported [51]. Although the latter study employed an aqueous dispersion of positively charged NDs, the silica surface is expected to be charged negatively at normal pH (IEP = 3.9, [50]) providing the same short-range electrostatic forces responsible for the ND surface self-assembly.

The EIP of SS304 surfaces, however, is somewhat acidic but could vary over a broader range from ca. 3.2 to 5.0 depending on the sample surface treatment according literature data summarized in [52]. Thus, at neutral pH or at a somewhat acidic pH of the DI water absorbing CO_2_ from air, the SS304 surface is expected to bare some negative change or no charge at all. Therefore, electrostatic interactions alone would not explain effects on −ND on tribological properties of SS304 surfaces.

One common feature observed in the data sets is that −ND dispersions produced through carboxylation consistently reduced the macroscopic friction coefficient relative to DI water by a factor of 2–5 for all stainless steel and alumina contacts studied and the value upon immersion consistently dropped into the range 0.05–0.1. QCM studies of stainless steel and alumina surfaces immersed in −ND dispersions meanwhile displayed behaviors consistent with a rapid uptake of −NDs, some slight but measurable increases in the surface roughness and distinct changes in the nature of the solid–liquid interfacial resistance to shear. These systems also exhibited an abrupt drop in *f* and an increase in *R* when re-exposed to pure DI water. The latter behavior is potentially explained by the suspended −ND nanoparticles acting as a lubricious slurry reducing resistance at the solid–liquid interface through potentially electrostatic repulsion with the rest of the −NDs in the surrounding suspension [20,43]. This suggestion, which is somewhat analogous to boundary lubrication and steric repulsion effects by mucinous glycoproteins boundary layers in aqueous biological settings [18], remains an intriguing possibility for the future investigations. The permanent changes observed in the surfaces morphology are likely associated with strong chemical attachment of −NDs in a manner distributed over the surface to form a more lubricous sliding interface than the bare surfaces alone.

It is notable that the literature on the contacts lubricated by aqueous ND suspensions for a range of materials reports the friction coefficients in the same range, 0.05–0.1, as observed here [2,7–8], while NDs similar to those employed here result in a markedly lower friction coefficients when treated with a dispersant so as to form colloids in oils [53]. This is consistent with a suggestion that the surface passivation treatments of NDs have great impact on the tribological properties. The notion is well known in the literature for diamond on diamond contacts in a variety of vacuum and humid environments [17,54–58]. The films of NDs strongly attached to surfaces are capable of providing both boundary lubrication and, potentially, a solid–liquid interface with a low resistance to shear. Therefore a custom design of nanolubrication systems by a proper chemical passivation of ND surfaces appears as a promising approach.

Hydroxylated NDs bearing a positive zeta potential in aquelus dispersion, produced no significant response in the frequency or resistance behavior of the QCM electrode covered with either alumina or SS304. While it is reasonable that the carboxylated −ND might exhibit more affinity to the alumina and stainless surfaces studied, one might conclude that the +ND suspensions would have little impact on the macroscale friction coefficients measured. But the friction increased significantly for the stainless steel materials. This may arise from corrosive and/or tribocorrosive effects at the macroscopic steel on steel interface being exacerbated by an abrasive action in the confined contact [59–60], a phenomena which was not probed by the AFM and QCM methods utilized here. It is notable that the changes in the QCM behavior upon immersion in +ND suspensions were very slow and gradual, in a stark contrast to the effects of the −ND suspensions. Detrimental wear at the macroscale might well out-pace any beneficial effects of ND for such liquid–solid interfaces.

We note that water was chosen as a liquid lubricant for this study so the results could be directly compared with preceding experiments of Liu et al. who employed QCM to investigate lubricating properties of aqueous suspensions of positively and negatively charged detonation nanodiamonds for gold electrode surfaces [9]. It is worthwhile to note that the methods described here are fully applicable to fluids other than water as long as the viscosity is sufficiently low for QCM to oscillate. Further studies will undoubtedly lead to a better understanding of third-body problems as well as improved design of the nanoparticle-based lubricants. Importantly, we have identified systems exhibiting beneficial, neutral, and detrimental tribology properties, facilitating additional experimental as well as theoretical studies from the first principles approach.

## Conclusion

A comparative study of the nanoscale and macroscale tribological attributes of alumina and stainless steel surfaces immersed in positively (hydroxylated) or negatively (carboxylated) charged nanodiamond (ND) dispersion is reported here. The work has revealed key similarities and differences between the surfaces that are effectively or ineffectively lubricated by aqueous suspensions of ND. The principle observations and conclusions are as follows:

- Immersion in −ND aqueous dispersion consistently resulted in a reduction in µ_k_, for the stainless steel and alumina samples studied, falling by a factor of 2–5 to a steady state value in the range 0.05–0.1.
- Immersion in +ND aqueous dispersion consistently increased µ_k_, for the stainless steel contacts and resulted in little to no increases for the alumina contacts.
- QCM and AFM measurements documented a rapid change in the surfaces of both alumina and stainless steel upon an exposure to −ND, consistent with a strong attachment of particles to the surfaces.
- The surfaces, upon uptake of ND, were characterized by a low resistance to shear at the interface between the solid and the aqueous −ND dispersion.
- Negligible polishing and/or abrasive effects were observed for QCM electrode surfaces after oscillating in either +ND or −ND dispersions for 1 h. The roughness of all the surfaces increased slightly upon an exposure to ND suspensions. This could be attributed to an attachment of NDs to the surface and/or some erosion effects.
- The +ND nanoparticles were not observed to rigidly adhere to the surfaces, for example, as a chemically bound adlayer, but some limited evidence was present for a loose attachment to alumina and very low coverages of dendritic aggregates on stainless steel.
- A suggested mechanism for the observations is that carboxylated −NDs in an aqueous dispersion form robust, lubricious film deposits on stainless steel and/or alumina surfaces that are both readily replenished by the surrounding suspensions and also glide through it with a relatively low resistance to shear, potentially because of the repulsive electrostatic forces between the individual particles.

In summary, this study provides for atomic scale details associated with systems that exhibit radically different macroscale tribological properties. It also reveals a broad class of materials that will be of great value in enabling theoretical efforts to predict and model complex lubricant interfaces.
